# Ca-Zn-Ag Alginate Aerogels for Wound Healing Applications: Swelling Behavior in Simulated Human Body Fluids and Effect on Macrophages

**DOI:** 10.3390/polym12112741

**Published:** 2020-11-18

**Authors:** Claudia Keil, Christopher Hübner, Constanze Richter, Sandy Lier, Lars Barthel, Vera Meyer, Raman Subrahmanyam, Pavel Gurikov, Irina Smirnova, Hajo Haase

**Affiliations:** 1 Department Food Chemistry and Toxicology, Institute of Food Technology and Food Chemistry, TU Berlin, Straße des 17. Juni 135, 10623 Berlin, Germany; c.huebner@tu-berlin.de (C.H.); constanze.richter@tu-berlin.de (C.R.); lier@campus.tu-berlin.de (S.L.); 2 Applied and Molecular Microbiology, Institute of Biotechnology, TU Berlin, Straße des 17. Juni 135, 10623 Berlin, Germany; lars.barthel@tu-berlin.de (L.B.); vera.meyer@tu-berlin.de (V.M.); 3 Institute of Thermal Separation Processes, Hamburg University of Technology, Eißendorfer Straße 38, 21073 Hamburg, Germany; raman.subrahmanyam@tuhh.de (R.S.); irina.smirnova@tuhh.de (I.S.); 4 Laboratory for Development and Modelling of Novel Nanoporous Materials, Hamburg University of Technology, Eißendorfer Straße 38, 21073 Hamburg, Germany; pavel.gurikov@tuhh.de

**Keywords:** aerogel, alginate, zinc, silver, wound dressing, albumin, antibacterial, Toll-like receptor, anti-inflammatory, nitric monoxide

## Abstract

Chronic non-healing wounds represent a substantial economic burden to healthcare systems and cause a considerable reduction in quality of life for those affected. Approximately 0.5–2% of the population in developed countries are projected to experience a chronic wound in their lifetime, necessitating further developments in the area of wound care materials. The use of aerogels for wound healing applications has increased due to their high exudate absorbency and ability to incorporate therapeutic substances, amongst them trace metals, to promote wound-healing. This study evaluates the swelling behavior of Ca-Zn-Ag-loaded alginate aerogels and their metal release upon incubation in human sweat or wound fluid substitutes. All aerogels show excellent liquid uptake from any of the formulas and high liquid holding capacities. Calcium is only marginally released into the swelling solvents, thus remaining as alginate bridging component aiding the absorption and fast transfer of liquids into the aerogel network. The zinc transfer quota is similar to those observed for common wound dressings in human and animal injury models. With respect to the immune regulatory function of zinc, cell culture studies show a high availability and anti-inflammatory activity of aerogel released Zn-species in RAW 264.7 macrophages. For silver, the balance between antibacterial effectiveness versus cytotoxicity remains a significant challenge for which the alginate aerogels need to be improved in the future. An increased knowledge of the transformations that alginate aerogels undergo in the course of the fabrication as well as during wound fluid exposure is necessary when aiming to create advanced, tissue-compatible aerogel products.

## 1. Introduction

Chronic wounds are a health issue with major impact on patients’ living quality and generate considerable costs for healthcare systems and societies [1,2]. It has been estimated that around 0.5 to 2% of the population in developed countries will experience a chronic wound during their lifetime [3,4]. Consequently, there is a need for developing improved wound care products. Recent predictions assume a rise in the global wound-closure products market exceeding $15 billion by 2022, accompanied by an accelerated progress for advanced textile and polymer materials in wound care [2]. Advanced wound care products combine (ideally multifactorial) characteristics of mechanical stability, biodegradability, debridement uptake and wound moistening ability as well as therapeutic potency via antimicrobial, immune modulatory or epidermal and dermal growth stimulatory activities [5].

The use of natural biopolymers and biocomposites opens a variety of options in the development of new wound dressing materials [6,7]. Promising biomatrices already employed in wound dressings are cellulose, starch, pectin or alginates [8,9]. Their transformation into highly porous aerogel materials [10,11,12,13,14] ameliorates their suitability for medical applications [15,16,17,18]. Polysaccharide hydrogels and their corresponding aerogels can be produced by covalent, ionic, pH-induced, thermal or cryo-cross linking, as well as a number of other methods [19,20,21,22]. A promising technology combining high material performances, ecological and safety aspects is a “green” carbon dioxide-facilitated processing, leading to a new generation of superabsorbent bioaerogels. Briefly, first an internal setting gelation technique utilizing pressurized CO_2_ is applied to enable controlled crosslinking of biopolymer chains through bivalent cation bridges (mostly Ca^2+^, Zn^2+^), forming a dynamic *egg*-*box*-like 3D hydrogel. These hydrogels are then converted into aerogels by a stepwise organic solvent exchange and supercritical carbon dioxide-assisted drying [12,23]. Of note, this procedure avoids using toxic compounds, which are inevitable for the synthesis of many synthetic polymeric aerogels [21]. As another advantage, wound active/reactive substances (e.g., antibiotics, antioxidants, immune-responsive drugs, growth factors, nanoparticles) can easily be embedded into the gel matrix or adsorbed onto the porous scaffold in the course of preparation [16,17,24,25,26]. These mediators are released into the wound environment during application, either through disintegration of the gel matrix or desorption from the aerogel pores [11,27] to speed up healing of acute or chronic inflamed wounds, a topic of great importance for both global healthcare systems and patients. Apart from their structural importance for polymer bridging, the choice of cations in the ionotropic gelation of carbohydrate polymers is relevant due to their beneficial impact on wound healing [28,29].

In a recent manuscript we reported the fabrication and metal analytics of alginate-based aerogels carrying calcium, zinc and silver cations. In addition, data on aerogel metal leaching into standard laboratory buffer solutions were provided [30]. These soluble metal species are effective antibacterial mediators, capable of defending the body against microbes/pathogens in addition to their impact on the various cell types in the healing wound [28,29,31]. Still, wound dressings intended for clinical trials require profound pretesting to assess their compatibility and bioactivity. In vitro tests, even though not fully recreating the in vivo wound pathophysiology or the interactions of the wound/peri-wound tissue and the wound dressing, allow an assessment of wound care products under controlled conditions, providing a first glance on how these materials affect the relevant cell types involved in wound healing.

This study investigates and compares swelling behavior and metal release from Ca-Zn-Ag-loaded alginate aerogels during a simulated application by utilizing ISO-standardized sweat or body fluid formulations designed to approximate the chemical environment of human plasma or wound fluid. Swelling supernatants were characterized with regard to their antimicrobial effectiveness. In addition, a macrophage cell model was used to assess their anti-inflammatory potency, focusing on the regulatory impact of swelling-derived soluble metal species on the Toll-like receptor 4-mediated innate immunity signaling cascade and the resulting synthesis of nitric monoxide.

## 2. Materials and Methods

### 2.1. Materials

AgNO_3_ (Sigma Aldrich, Munich, Germany, CatNr 204390); antibodies: anti-ERK1/2 (Cell Signaling, Leiden, Netherlands, CatNr 4695), anti-Thr202/Tyr204-phosphorylated-ERK1/2 (Cell Signaling, Leiden, Netherlands, CatNr 9101), goat anti-rabbit IgG-HRP (Santa Cruz Biotechnology, Dallas, TX, USA; CatNr sc-2357); Aprotonin (Sigma Aldrich, Munich, Germany, CatNr A6103); Bicinchoninic acid (BCA (Sigma Aldrich, Munich, Germany, CatNr B9643); bovine serum albumin (BSA) (Sigma Aldrich, Munich, Germany; CatNr 05470); CaCl_2_ (Carl Roth, Karlsruhe, Germany, CatNr CN92.2);Dulbeccos modified Eagles medium (DMEM) (PAN-Biotech, Aidenbach, Germany, CatNr P04-04500); dry milk powder (Carl Roth, Karlsruhe, Germany, CatNr T145.3); Ethylenediaminetetraacetic acid (EDTA) (Sigma Aldrich, Munich, Germany, CatNr 431788); Ethylene Glycol Tetraacetic Acid (EGTA) (Sigma Aldrich, Munich, Germany, CatNr 03777); fetal calf serum (FCS) (CCPro, Oberdorla, Germany; CatNr S-10-L); glucose (Carl Roth, Karlsruhe, Germany, CatNr X997.2); HEPES (Carl Roth, Karlsruhe, Germany, CatNr 9105.3); glycerol (Carl Roth, Karlsruhe, Germany, CatNr 6967.1); L-Histidine (Sigma Aldrich, Munich, Germany; CatNr H8000); KCl (Sigma Aldrich, Munich, Germany; CatNr P9541); K_2_HPO_4_ (Carl Roth, Karlsruhe, Germany, CatNr P749.2); 3-(4,5-dimethylthiazol-2-yl)-2,5-diphenyltetrazolium bromide (MTT) (Carl Roth, Karlsruhe, Germany, CatNr 4022.2); Lipopolysaccharides from Escherichia coli (LPS_E. coli_) (Sigma Aldrich, Munich, Germany, CatNr L4130); MgCl_2_ (Carl Roth, Karlsruhe, Germany, CatNr KK36.3); NaCl (Carl Roth, Karlsruhe, Germany, CatNr 3957.2); NaDeoxycholat (Sigma Aldrich, Munich, Germany; CatNr D6750); NaH_2_PO_4_ (Carl Roth, Karlsruhe, Germany, CatNr T878.3); NaHCO_3_ (Carl Roth, Karlsruhe, Germany, CatNr 6885.1); NaF (Sigma Aldrich, Munich, Germany; CatNr 215309); NaNO_2_ (Carl Roth, Karlsruhe, Germany, CatNr 8604.1); Na_2_SO_4_ (Carl Roth, Karlsruhe, Germany, CatNr 0966.2); Na_4_P_2_O_7_ (Sigma Aldrich, Munich, Germany, CatNr P8010); Na_3_VO_4_ (Sigma Aldrich, Munich, Germany, CatNr 450243); NaC_12_H_25_SO_4_ (SDS) (Carl Roth, Karlsruhe, Germany, CatNr 2326.3); N-(1-naphtyl) ethylenediamine dihydrochloride (Carl Roth, Karlsruhe, Germany, CatNr 4342.2); nitrocellulose membrane (Bio-Rad Laboratories GmbH, Feldkirchen, Germany, CatNr 1620115); penicillin-streptomycin (Sigma Aldrich, Munich, Germany, CatNr P4333); phenylmethylsulfonyl fluoride (PMSF) (Sigma Aldrich, Munich, Germany, CatNr 78830); ponceau S (Sigma Aldrich, Munich, Germany, CatNr P3504); sulfanilamide (Carl Roth, Karlsruhe, Germany, CatNr 4716.1); Tris (Carl Roth, Karlsruhe, Germany, CatNr 5429.2); Triton X-100 (Carl Roth, Karlsruhe, Germany, CatNr 3051.4); Tween 20 (Carl Roth, Karlsruhe, Germany, CatNr 9127.2); West Pico PLUS Chemiluminescent Substrate (ThermoFisher, Waltham, MA, USA, CatNr 34579); Zinpyr-1 (Biomol GmbH, Hamburg, Germany, CatNr CDX-Z0001); ZnSO_4_·7H_2_O (Sigma Aldrich, Munich, Germany; ZnO (Merck, Darmstadt, Germany, CatNr 2643836). All other chemicals were purchased from standard sources.

### 2.2. Aerogel Preparation

Synthesis and characterization of the aerogel particles used within this study (Table 1) is described in a previous paper [30]. Briefly 1 wt% sodium alginate solutions were mixed with cross linkers in a defined cation to alginate ratio to achieve cross-linking degrees of either Q1 (1.8 mmol_Cation_/g_dry alginate_) or Q0.5 (0.9 mmol_Cation_/g_dry alginate_). In case of Ca-Zn-Ag aerogels AgNO_3_ was admixed into the gelling solution in a total amount of 0.91 mmol_AgNO3_/g_dry alginate_. Subsequently aerogels were prepared via stepwise CO_2_- induced internal setting gelation, solvent exchange and supercritical carbon dioxide-assisted drying [12]. The aerogels were subsequently stored in the dark at 4 °C in closed thermoplastic containers.

### 2.3. Aerogel Swelling Studies

Aerogel swelling was done as described previously [30].Briefly, aerogels were incubated in a final concentration of 10 mg/mL in either synthetic sweat solution ISO 5.5 (85.5 mM NaCl; 3.22 mM L-Histidine; 18.3 mM NaH_2_PO_4_; pH 5.5) or ISO 8.0 (85.5 mM NaCl; 3.22 mM L-Histidine; 35.2 mM Na_2_HPO_4_; pH 8.0) [32], simulated body fluid (SBF: 137 mM NaCl; 4.1 mM NaHCO_3_; 3 mM KCl; 0.1 mM K_2_HPO_4_; 1.5 mM MgCl_2_; 2.5 mM CaCl_2_; 0.5 mM Na_2_SO_4_; 50 mM Tris; 45 mM HCl; pH 7.4) [33] or albumin-enriched fluid (simulated body fluid + 1.8% (*w*/*v*) BSA (SBF-albumin)). Following the swelling period (5 min), the supernatants were completely removed and subjected to metal analytics (flame atomic absorption spectrometry; Perkin Elmer AAnalyst 800; Perkin Elmer, Rodgau, Germany) to estimate metal release [30]. SBF-albumin swelling supernatants were further applied in antibacterial tests or cell culture experiments. The remaining gels were weighed and liquid uptake (LU) determined from the weight of the swollen sample (m_s_) and the initial weight (m_i_) with LU= (m_s −_ m_i_) × 100/m_i_ [13].

### 2.4. Serum Albumin Binding Assay

To evaluate albumin binding capacity, the aerogel swelling procedure was performed in SBF-albumin fluid. The remaining levels of protein in the swelling supernatant were measured by using the bicinchoninic acid (BCA) assay [34].

### 2.5. Antibacterial Test

Antimicrobial effectiveness was studied by measuring the effect of SBF-albumin swelling supernatants on growth (OD_600_ cell density) and viability (MTT assay; reduction of the yellow tetrazolium salt (3-(4,5-dimethylthiazol-2-yl)-2,5-diphenyltetrazolium bromide) of *Escherichia coli* strain BL21(D3) and *Staphylococcus warneri* (strain dsm-20316) [35]. Briefly, overnight cultures of bacteria were diluted 1:250 in their respective culture media and grown in 96-well plate cavities in the presence of aerogel swelling supernatans or metal salt solutions for 4 h. OD600 bacterial density was measured on an Infinite M200 microplate reader (Tecan, Crailsheim, Germany). Subsequently, bacteria were treated with 0.1 mg/l MTT in culture medium following 30 min lysis in isopropanol and determination of the formazan absorption at 570 nm with a reference wavelength of 630 nm (Tecan, Crailsheim, Germany). Incubations with aerogel-untreated SBF-albumin were included to define the maximum possible bacterial growth and metabolic activity (100% control).

### 2.6. Cell Culture 

RAW 264.7 macrophages [36] were used as cell model to examine the immune modulatory properties of aerogel swelling supernatants. Cells were grown in DMEM containing 10% FCS (heat inactivated for 30 min at 56 °C), 2 mM l-glutamine, 100 µg/mL potassium penicillin, and 100 µg/mL streptomycin. 

### 2.7. Zn^2+^ Measurement with Fluorescent Probes

Intracellular free zinc measurement was performed with a slightly modified from a protocol published before [37]. Briefly, cells were grown in 96-well plate cavities up to 70–75% confluency before loading with 2.5 µM Zinpyr-1 in a loading buffer (10 mM HEPES; pH 7.35; 120 mM NaCl; 5.4 mM KCl; 5 mM glucose; 1.3 mM CaCl_2_; 1 mM MgCl_2_; 1 mM NaH_2_PO_4_; 0.3% (*w/v)* BSA). Next, aerogel swelling supernatans or metal salt solutions (ZnSO_4_, ZnO, CaCl_2_, AgNO_3_) were added and fluorescence was measured after 30 min incubation on a Tecan Infinite M200 reader using excitation/emission wavelengths of 492/527 nm for Zinpyr-1 [38]. In addition, confocal laser scanning microscopy (Leica TCS SP8 CLMS equipped with LAS X 3.5.5.19976 software platform; Leica, Wetzlar, Germany, using a HC PL APO CS2 63x/1.20 water objective; filters settings were λ_Exc_ 488 nm/ λ_Em_ 500–550 nm) was performed to monitor cellular distribution of Zinpyr-1-accessible zinc. 

### 2.8. Griess Assay

Stimulation of Raw 264.7 cells with bacterial lipopolysaccharides (LPS) triggers the production of the antimicrobial effector nitric monoxide (NO^•^). Its stable metabolite nitrite can be detected following reaction with the Griess reagent [37]. Briefly, cells were grown in 96-well plate cavities up to 70–75% confluence before 24 h stimulation with 100 ng/mL *Escherichia coli* LPS or LPS together with either 50 µl aerogel supernatants or metal salt solutions in a total volume of 200 µl growth medium. Afterwards cell supernatants were collected, and cell layers used for viability measurements applying 0.25 mg/mL MTT. Cell supernatants from viable cells were analyzed for NO^●^ content by the Griess method [39]. In brief, 50 µl of cell supernatant was mixed with 50 µl of 1% sulfanilamide (dissolved in 5% H_3_PO_4_) for 10 min. 50 µl 0.1% *N-*(1-naphtyl) ethylenediamine dihydrochloride solution was added and reactions incubated for another 10 min in the dark. Nitrite production was determined photometrically at 520 nm in a Tecan Infinite M200 reader and quantified based on a standard curve from 0.78 to 50 µM sodium nitrite.

### 2.9. Western Blotting

RAW 264.7 cells grown in 24-well plate cavities (seeding density 7 × 10^5^/500 µl DMEM) were stimulated with freeze-inactivated *Escherichia coli* BL21(D3) either in the presence or absence of aerogel supernatants or metal salt solution for 30 min. Supernatants were removed, cells lysed in sample buffer (10 mM Tris [pH 7.4]; 100 mM NaCl; 1 mM EDTA; 1 mM EGTA; 1 mM NaF; 20 mM Na_4_P_2_O_7_; 2 mM Na_3_VO_4_; 1 mM PMSF; 2 µg/mL aprotinin; 0.1% (*w*/*v)* SDS; 1% (*v*/*v*) Triton X-100; 10% (*v*/*v*) glycerol; 0.25% (*w*/*v)* NaDeoxycholat) and samples were sonicated before separation of proteins by denaturing SDS-PAGE and blotting to nitrocellulose membranes. Uniform loading of gels was confirmed by staining with Ponceau S. After destaining, membranes were blocked for 1 h with TBST (20 mM Tris-HCl [pH 7.6]; 136 mM NaCl; 0.1% (*v*/*v*) Tween 20) containing 5% fat-free dry milk and incubated overnight with primary antibodies directed against total-ERK1/2 or Thr202/Tyr204-phosphorylated-ERK1/2 diluted in TBST containing 5% BSA. Subsequently, membranes were washed three times with 25 mL TBS-T and incubated for 1 h with goat anti-rabbit-HRP followed by detection with West Pico PLUS Chemiluminescent Substrate on a LAS-3000 (Fuji Photo Film (Europe) GmbH, Duesseldorf, Germany) image reader [40]. Densitometric quantification was performed with ImageJ software [41].

### 2.10. Statistical Analyses

The data shown are based on three independently performed experiments. The statistical significance of the experimental results was calculated with GraphPad prism software version 8.02 (GraphPad Software Inc., San Diego, CA, USA) using the tests indicated in the respective figure legends.

## 3. Results and Discussion

### 3.1. Aerogel Swelling Behavior and Metal Release

**Figure 1:**
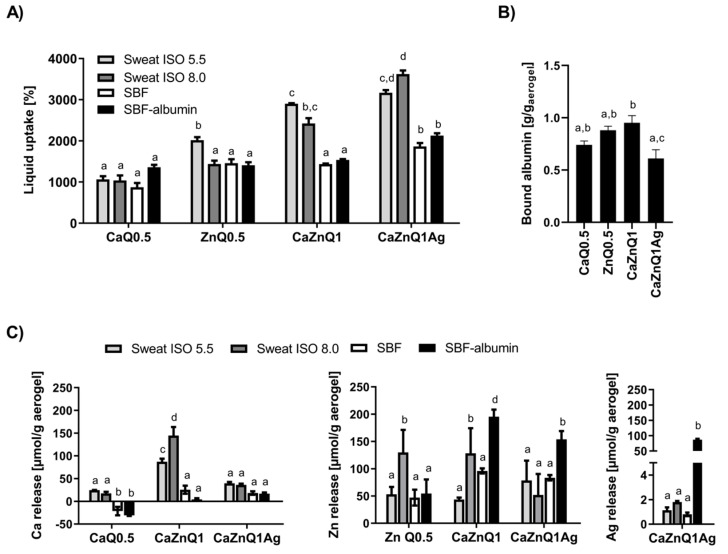
Swelling behavior and metal release of alginate-based aerogels in substitutes for human body fluids. Aerogels were incubated for 5 min in the body fluid substitutes before quantification of (**A**) liquid uptake (**B**) albumin binding and (**C**) metal release. Data are presented as means ± SEM of three independent experiments. Bars sharing letters are not significantly different (repeated measures ANOVA with Tukey’s multiple comparisons post-hoc test).

Liquid uptake and metal release from the Ca-Zn-Ag aerogels was previously studied using bidistilled water and a HEPES-based buffer for swelling [30]. However, for medical applications a pre-clinical assessment under conditions closer to an intact or wounded human skin milieu is required. Therefore, we re-assessed these parameters applying (1) ISO-standardized sweat formulations [42] mimicking the composition and pH of human perspirations, (2) a simulated body fluid (SBF) with ion concentrations equal to those of human blood plasma [33], and (3) a SBF with added albumin, as this is the most abundant blood plasma protein (accounting for ~60% of human blood proteins, ~0.53–0.75 mM) [43]. Moreover, albumin is released into wound fluids, in particular during inflammation where increased capillary permeability allows leakage of this protein into the extravascular space [44]. As shown in Figure 1A, the aerogels are superabsorbers from any of the selected formulations. The open-porous structure and high surface area of these alginate aerogels [30] promotes rapid liquid uptake from any of the investigated human body fluid substitutes within a previously established time frame sufficient for achieving maximum swelling of the aerogels [30]. We anticipate the water vapor permeability of the alginate-based gels to be in the range of common calcium alginate wound dressings (0.00001–0.00002 kg/m.Pa.s) since the chemical structure of the calcium alginate aerogel does not vary significantly [45].

Khattab et al. [46] recently reported a cellulose-based liquid-stretchable aerogel sensor suitable for assessment of skin sweat status. This study also identified alginate aerogels as promising ingredients in wound dressings or cosmetic products to trap human sebum and perspirations. From a medical perspective the perspiration-soaking ability of Ca-Zn-Ag aerogels is advantageous when aiming to restrict moisture lesions in skin and associated periwound skin damage. In this respect, the multication-loaded alginate aerogels seem to be particularly well suited for uptake of sweat-mimicking fluids (Figure 1A), but more comprehensive work is required to understand the underlying aspect of swelling and liquid uptake from perspirations in detail.

The liquid uptake of synthetic sweat formulas and SBFs was distinctly lower than the quota determined for bidistilled water as swelling agent (~4000–7000%, [30]). Presumably, a disintegration of the alginate aerogel occurs following treatment with human body fluid substitutes. These contain a considerable amount of monovalent ions (Na^+^, K^+^) and phosphates, displacing the bivalent bridging cations from the egg-box cavity. Such disintegration was previously reported for several other alginate aerogels [24,47,48,49]. Cation release varied substantially depending on the swelling solution as well as the investigated cation (Figure 1C). Upon wetting with SBF, zinc was more efficiently released from the alginate aerogels than calcium. This was also observed after swelling of these aerogels in Na^+^-enriched HEPES, confirming the weaker attachment of zinc to the alginate gel [30,50].

In physiological environments such as human plasma or wound fluids, the aerogels will be surrounded by a protein corona that may alter their surface and porosity. The results of the protein binding assay revealed the highest albumin adsorption onto CaZnQ1 aerogels (Figure 1B), probably because of their higher surface area (Table 1). Approximately 60% of the BSA contained in the SBF-albumin was bound by the CaZnQ1 aerogel. In blood, albumin is crucial for binding, storage and transport of zinc. Almost 80% of total plasma zinc (~14 µM) is thought to be bound to human albumin, constituting the bulk of the potentially exchangeable plasma zinc pool [43,51]. However, under normal conditions only 2% of the circulating albumin molecules carry a zinc ion [52], leaving a vast capacity for accepting zinc from topically applied wound dressings (such as Ca-Zn-Ag aerogels). Here the presence of albumin enhanced zinc release from alginate aerogels over SBF solutions with no protein (Figure 1C). Thus, it is quite likely that systemic albumin will also act as a zinc-carrier in the wounded tissue. The calcium affinity of human albumin is rather low (K_d_ = 0.67 mM [43]). Hence, BSA only marginally contributes to calcium release from alginate aerogels into SBF-albumin (Figure 1C). Regarding clinical applications the total calcium and zinc concentrations in any of the aerogel SBF-albumin swelling concentrations (Figure S1) were close to those determined in sera from skin-injured humans and animals treated with Ca/Zn-ion impregnated wound dressings [29,53,54]. The considerable silver release from the CaZnQ1Ag aerogels into the SBF-albumin solution (Figure 1C, Supplementary Figure S1) is a matter of concern. Silver levels in the swelling supernatants were orders of magnitude higher than those measured in wound fluids or sera from patients topically treated with Ag-coated foam dressing Acticoat or nitrate silver sulfadiazine (~ 2–10 µM Ag in these patients’ sera) [55,56]. Local toxicity or argyrosis upon topic treatment with CaZnQ1Ag aerogels have to be considered, necessitating fine tuning of the silver quantity released by the alginate aerogels.

### 3.2. Antibacterial Effectiveness of Aerogel Swelling Supernatants

**Figure 2:**
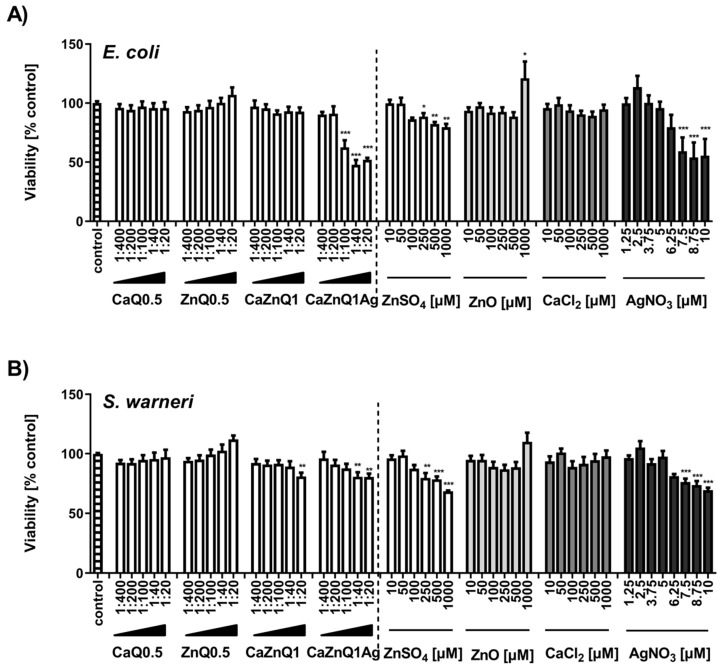
Effect of aerogel soluble mediators on bacterial growth. Growth of *E. coli* (**A**) or *S. warneri* (**B**) after 3h culture in the presence of aerogel swelling supernatant dilutions or metal salt solutions generated in SBF-albumin was analyzed by measuring the optical density at 600 nm. A viability of 100% corresponds to the signal obtained with bacteria in the presence of untreated SBF-albumin. The designations on the X axes state the dilution of aerogel supernatants and the final concentration of metal salts within the incubations. Data are presented as means ± SEM of three independent experiments. Statistically significant differences from control are marked by asterisks (* *p* < 0.05, ** *p* < 0.01, *** *p* < 0.001; One-Way ANOVA with Tukey’s Multiple Comparison Test).

*Escherichia coli* and *Staphylococcus warneri* were selected as representatives for gram-positive and gram-negative bacteria. These species represent relevant opportunistic or nosocomial pathogens in wounded tissues [57,58]. The turbidity measurement of microbial cultures revealed very limited bactericidal effectiveness of serial dilutions of SBF-albumin swelling supernatants from Ca and/or Zn loaded aerogels. Likewise, application of inorganic zinc and calcium salts showed almost no toxicity (Figure 2A,B). For CaZnQ1Ag a concentration-dependent effect was observed with a greater impact on *Escherichia coli*. Based on the estimated amount of silver in the SBF-albumin swelling supernatants (~1700 µM; Supplementary Figure S1) the actual effectiveness was lower compared to AgNO_3_, suggesting a different speciation of silver released from the aerogel, altering its effect, e.g., by affecting its availability for bacteria (Figure 2A,B). Still the total quantity of silver within the undiluted CaZnQ1Ag swelling concentrate delivers silver far in excess of bactericidal concentrations [35,59].

**Figure 3:**
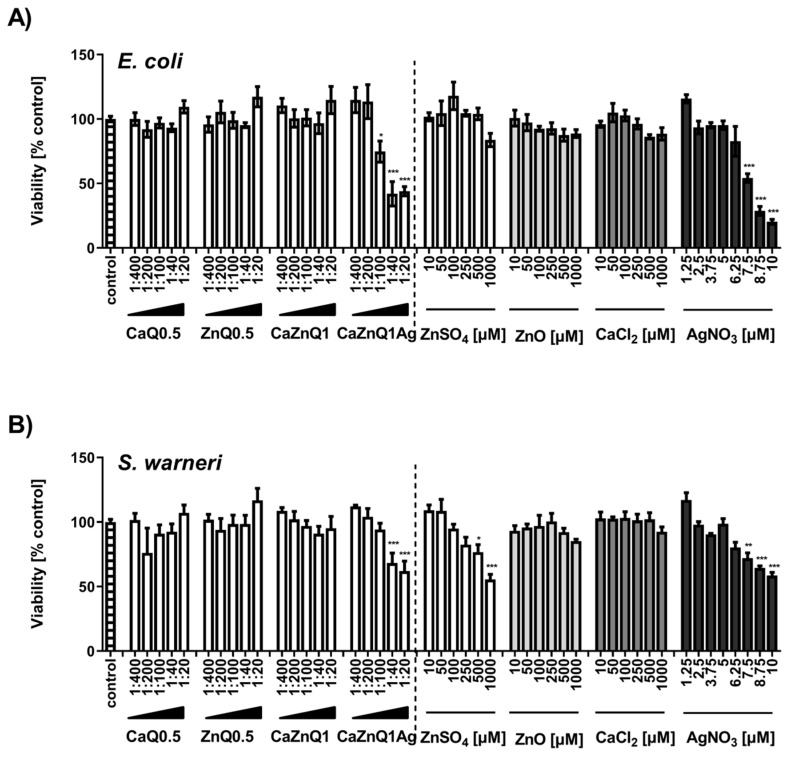
Effect of aerogel soluble mediators on bacterial metabolic activity. Viability of *E. coli* (**A**) or *S. warneri* (**B**) after 3h culture in the presence of aerogel swelling supernatant dilutions or metal salt solutions generated in SBF-albumin was analyzed by MTT assay. A viability of 100% corresponds to the signal obtained with bacteria in the presence of untreated SBF-albumin. The designations on the X axes state the dilution of aerogel gel supernatants and the final concentration of metal salts within the incubations. Data are presented as means ± SEM of three independent experiments. Statistically significant differences from control are marked by asterisks (* *p* < 0.05, ** *p* < 0.01, *** *p* <0.001; One-Way ANOVA with Tukey’s Multiple Comparison Test).

In principle, determination of bacterial metabolic activity with the MTT assay confirms the results of the OD_600_ assay. AgNO_3_ and the CaZnQ1Ag swelling supernatant were particularly effective in inhibiting bacterial metabolism (Figure 3A,B). For the CaZnQ1Ag aerogel-soluble mediators a maximum bacterial metabolic inhibitory effect of ~ 50% was observed, where the least diluted 1:20 and 1:40 samples were comparable in antibacterial efficacy within the experimental fluctuation margins. ZnO showed no statistically relevant antibacterial efficacy against *Escherichia coli* and *Staphylococcus warneri* over the entire concentration range when evaluating the MTT-reducing activity (Figure 3A,B). We attribute the slight increase in *E. coli* OD600 density following high ZnO treatment (1000 µM; Figure 2A) to the assembly of ZnO particles in bacteria enriched growth media. ZnO-related increase in turbidity was not observed in media in the absence of bacteria, which we included as negative controls.

Bacterial burden is believed to play an extensive role in impaired healing of chronic wounds and the development of infection-related complications. Yet, it has to be noted that the present study aimed to evaluate, in particular, the antibacterial effectiveness of the aerogel soluble metal fractions. Still, functionality of the aerogels in real wound situations is also affected by intermolecular interactions between bacteria and gel surfaces [60,61] in the local human skin milieu. These interactions would be identifiable in an *ex-vivo* human skin model [62], followed by application on animals and finally human volunteers in clinical trials.

### 3.3. Immune Modulatory Potency of Aerogel Swelling Supernatants

**Figure 4:**
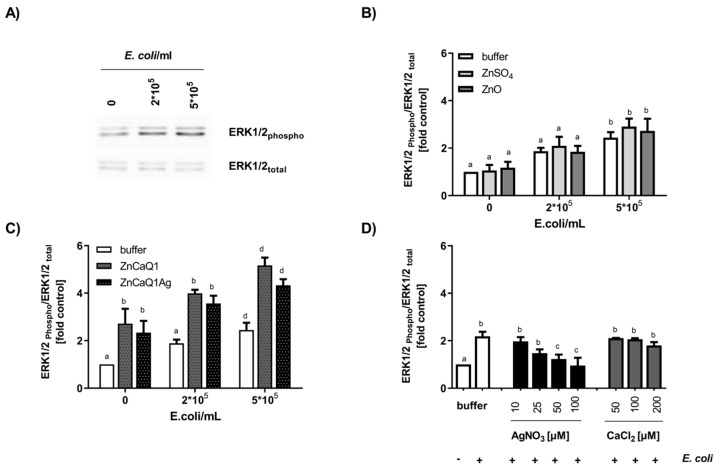
Effect of aerogel swelling supernatants on TLR4-induced ERK1/2 phosphorylation in RAW 264.7 cells. Macrophages were stimulated with *E. coli* for 30 min in the presence or absence of aerogel swelling supernatants or metal salt solutions generated in SBF-albumin. Whole cell lysates were separated by SDS-PAGE and subjected to Western blot detection applying antibodies against phosphorylated or total ERK1/2. (**A**) Representative Western blot images of phosphorylated-ERK and total ERK protein (**B**,**C**) Aerogel swelling supernatants and zinc solutions were applied with a final zinc concentration of 100 µM. (**D**) Effect of AgNO_3_ or CaCl_2_ on *E. coli*-induced ERK1/2 phosphorylation. Data are presented as means ± SEM of three independent experiments. (Bars sharing letters are not significantly different; repeated measures ANOVA with Tukey’s multiple comparisons post-hoc test).

**Figure 5:**
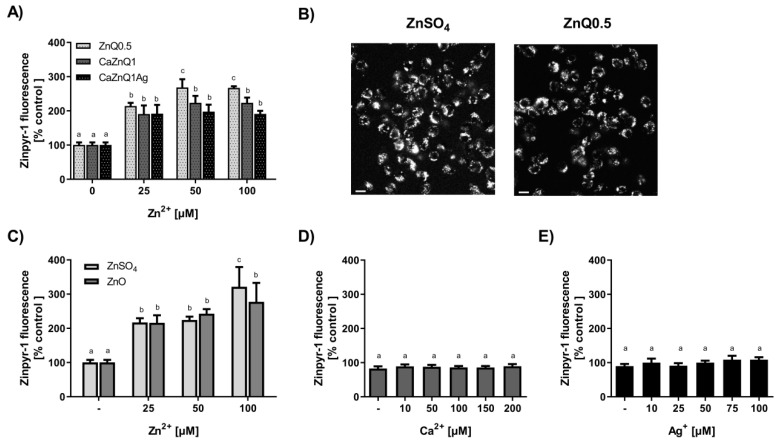
Determination of intracellular free zinc in RAW 264.7 cells. Cells were loaded with the zinc sensor Zinpyr-1 before addition of supernatants from aerogels or metal salt solutions generated in SBF-albumin. Quantitative changes in cellular Zinpyr-1 fluorescence (**A**,**C**–**E**) as well as fluorescence microscopic pictures (scale bar: 50 µm) (**B**) are shown. The designations on the X axes state the final zinc (**A**,**C**), calcium (**D**) or silver (**E**) concentrations within the incubations. Quantitative data are shown as means ± SEM of at least three independent experiments (Bars sharing letters are not significantly different; repeated measures ANOVA with Tukey’s multiple comparisons post-hoc test).

Macrophages as part of the innate immune system are crucial for wound healing. They initiate an inflammatory response immediately upon wounding, which can trigger infiltration of neutrophils and thus accelerate bacterial clearance. However, prolonged inflammation is detrimental and can contribute to chronification of wounds and healing difficulties [63]. The sensing of infection and strengthening of innate immunity by macrophages are mediated by pattern recognition receptors, including Toll-like receptor TLR4 [64]. TLR4 complexes, localized on the cell surface of macrophages, can register intact bacteria as well as isolated bacterial LPS. Sensing of pathogens by this receptor drives intracellular signaling cascades mediating the production of inflammatory factors. Here the activation status of the MAPK- pathway (MAPK= mitogen-activated protein kinase) is decisive with regard to the quantities of inflammatory mediators produced. As part of the MAPK-pathway, the protein kinase ERK1/2 is regulated in a phosphorylation-dependent manner. Thus, covalent phosphate attachment to ERK1/2 protein leads to increased kinase activity while dephosphorylation restores baseline activity [37,65]. Previous studies revealed a regulatory role of intracellular free zinc ions on TLR4-signaling with zinc augmenting ERK1/2 phosphorylation in macrophages [37,40,66]. These observations led us to evaluate the modulatory potency of zinc-containing aerogel swelling supernatants in cultivated RAW 264.7 macrophages, focusing on TLR4 signaling. Western blot experiments were performed to analyze RAW 264.7 ERK1/2 phosphorylation upon stimulation with *E. coli* suspensions in the absence or presence of aerogel swelling supernatants (Figure 4). Applying two different antibodies specific for either total ERK1/2 or phosphorylated ERK1/2 (Thr202/Tyr204) allowed monitoring the overall amount of cellular ERK1/2 and also its phosphorylation status [37,40]. In addition, Zinpyr-1, a membrane-permeant fluorescent sensor with a high affinity for Zn (K_d_ = 0.7 ± 0.1 nM; [38]), but insensitive to calcium and silver [67], was used for the investigation of intracellular free zinc (Figure 5).

TLR4 stimulation with *E. coli* dose-dependently increased phosphorylation of ERK1/2 (Figure 4A,B). In case of co-incubation with bacteria and aerogel CaZnQ1 or CaZnQ1Ag swelling supernatants diluted to a final zinc concentration of 100 µM, ERK1/2 phosphorylation was increased. A CaCl_2_ bonus up to 200 µM added to the DMEM medium (basal calcium concentration ~1.3 mM) had no impact on this TLR4-dependent signaling pathway (Figure 4D). The calcium input from the SBF-albumin aerogel supernatants was ~150 µM (calculated from the metal concentration of undiluted supernatant shown in Figure S1). For AgNO_3_ a dose-dependent inhibitory effect on ERK1/2 phosphorylation was observed (Figure 4D), yet silver delivered from the CaZnQ1Ag swelling supernatant (final concentration of ~17 µM, based on data in Figure S1) was below the effectual dose (Figure 4D). Thus, effects of the aerogel SBF-albumin mobile fraction on ERK1/2 phosphorylation are predominantly attributable to zinc species that affect the TLR4 cascade either via extracellular activation of the ZnR/GPR39 G-protein coupled receptor [68] or via intracellular zincergic signaling. An intracellular regulatory mechanism is supported by the estimation of the cellular free zinc levels (Figure 5A–C), showing almost comparable increase in Zinpyr-1 fluorescence upon incubation of RAW 264.7 with either Zn-aerogel swelling supernatant solutions or ZnSO_4_/ZnO, suggesting comparable zinc availability.

In direct comparison to inorganic zinc sources (ZnSO_4_/ZnO), RAW 264.7 macrophages responded with stronger ERK1/2 phosphorylation to aerogel swelling supernatants containing the same amount of zinc ions (Figure 4B,C). This leads to the assumption that aerogel alginate backbone components induce TLR4 or other ERK-dependent signaling pathways, either by acting as a ligand for plasma membrane receptors or affecting their downstream signaling pathways. This hypothesis is consistent with the observation of higher ERK1/2 baseline phosphorylation for aerogel swelling supernatant treatment in the absence of *E. coli* (Figure 4C). In accordance, Fang et al. [69] showed the induction of TLR4-signaling by alginate-derived guluronate oligosaccharide in RAW 264.7 macrophages.

**Figure 6:**
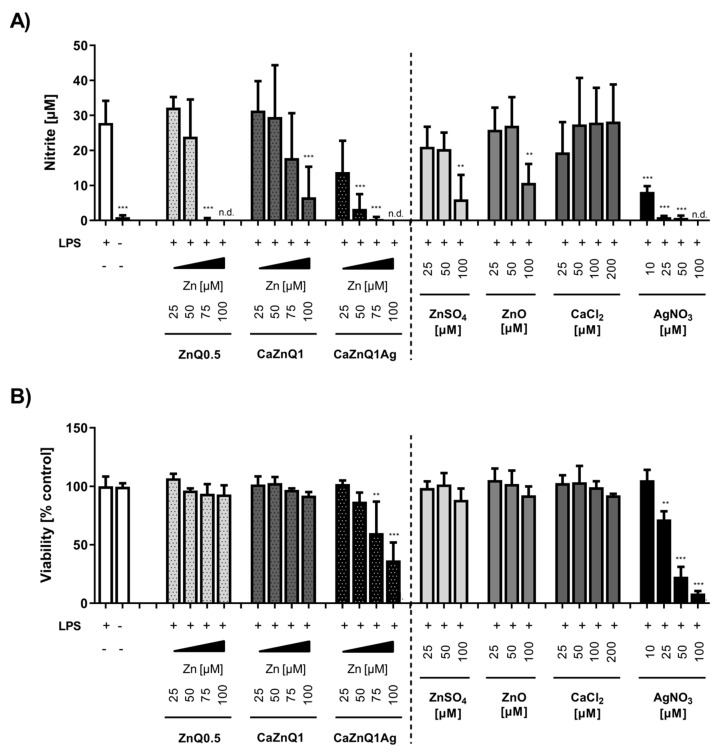
Impact of aerogel swelling supernatants on LPS-induced nitric monoxide production in RAW 264.7 cells. Supernatants from aerogels or metal salt solutions generated in SBF-albumin were applied on RAW 264.7 macrophage cells to study their impact on bacterial LPS-induced NO^●^ production, measured by its stable degradation product nitrite (**A**), and the corresponding viability (**B**) of RAW 264.7 cells. The designations on the X axes state the final amounts of aerogel-derived zinc and the final concentration of metal salts within the incubations. n.d. = not detected. Data are presented as means ± SEM of three independent experiments. Statistically significant differences from LPS-treatment (**A**) or control (**B**) are marked by asterisks (** *p* < 0.01, *** *p* < 0.001; One-Way ANOVA with Tukey’s Multiple Comparison Test).

TLR4 signaling in macrophages induces the production of many pro-inflammatory molecules, amongst them the effector nitric monoxide. NO^•^ plays a critical role in the wound-healing process. It is important as a noxious defense molecule against infectious organisms, mediates vasodilation in blood vessels and also coordinates host immune cells, thereby locally controlling the immune response [70]. RAW 264.7 cells activated with *E. coli* LPS for 24 h generated around 28 µM of nitrite, almost the same amount as in previous studies [71,72]. Zinc orchestrates and regulates the inflammatory balance within the local tissue environment [73]. Swelling supernatants obtained from ZnQ0.5 aerogels dose-dependently decreased LPS-induced nitrite concentrations in cell supernatants. This was not due to cytotoxicity, because viability was not affected by the ZnQ0.5 swelling supernatant under these conditions (Figure 6A,B). The influence on TLR4-induced NO^•^ release was comparable in efficiency to inorganic zinc (Figure 6B). The anti- inflammatory potency of the Zn alginate aerogels provided by the wound fluid-accessible zinc fraction could be advantageous for the treatment of wounds showing signs of chronic inflammation and delayed healing. On the other hand, the application of the CaZnQ1Ag aerogels has to be viewed much more carefully, as indications of silver cytotoxicity were found in the cell culture studies upon prolonged incubation (Figure 6B). Similarly, in a recently published in vitro screening of clinically applied wound dressings, the silver alginate Biatain^®^ scored rather poorly in cytocompatibility [74]. Very likely the amount of silver released by the alginate wound pad overstrained the cellular buffering capacity, thus damaging the cells irreversibly.

## 4. Conclusions

Alginate aerogels augmented with multiple cations can provide the next generation of superabsorbent medical devices for advanced wound care. This work shows that exposure of Ca-Zn-Ag alginate aerogels to body fluid formulations designed to match the chemical composition of human sweat or wound fluids led to substantial metal transfer into the supernatants. Calcium was only moderately released into any of the swelling solvents, thus remaining as alginate bridging component aiding the absorbency and fast transfer of liquids into the aerogel network. Zinc release into wound fluid-like liquids was close to those observed for common zinc ion-impregnated wound care products in human and animal injury models. The zinc-species within these swelling supernatants were able to modulate inflammatory signaling pathways in macrophages, with an anti-inflammatory outcome similar to inorganic zinc sources (ZnSO_4_/ZnO). For silver, the balance between antibacterial effectiveness versus cytotoxicity remains a significant challenge for which the alginate aerogels need to be improved in the future. Based on the results found for macrophages, an in-depth analysis of the multi-cation-loaded alginate aerogels on other cell types involved in the wound healing process is required prior to preclinical testing on animals or human volunteers. Additionally, the aspect of wound material sterilization needs to be considered. Physical treatments, such as plasma processing, may generate reactive oxygen species leading to the depolymerization of the alginate polysaccharide chains, thereby impacting swelling properties and release of bioactive soluble metal mediators from the aerogels. Furthermore, the long-term stability of alginate-based aerogels under conditions recommended by ICH (International Council for Harmonization of Technical Requirements for Pharmaceuticals for Human Use) has to be checked as there are no data available in the literature up to now. Increased knowledge of the transformations that alginates undergo in the course of the wound material fabrication, storage as well as during wound fluid exposure is necessary when aiming to create advanced, tissue compatible aerogel products.

## Figures and Tables

**Table 1:** Characteristics of the aerogel particles.

	Initial Alginate Dispersion mmol_Cation_/g_dry alginate_			Aerogels			
	Ca	Zn	Ag	Surface Area m^2^/g	mmol Ca/g	mmol Zn/g	mmol Ag/g
**CaQ0.5**	0.9	-	-	499	0.705 ± 0.093	0.001 ± 0.001	n.d.
**ZnQ0.5**	-	0.9	-	361	0.035 ± 0.007	0.156 ± 0.080	n.d.
**CaZnQ1**	0.9	0.9	-	617	0.774 ± 0.083	0.112 ± 0.033	n.d.
**CaZnQ1Ag**	0.9	0.9	0.91	475	0.743 ± 0.056	0.270 ± 0.058	0.569 ± 0.031

Data previously published in [30]. n.d. = not detected.

## Supplementary Materials

The following are available online at https://www.mdpi.com/2073-4360/12/11/2741/s1, Figure S1: Quantification of the total metal concentrations in the aerogel swelling supernatants.
