# Supplementary material for: Ca-Zn-Ag Alginate Aerogels for Wound Healing Applications: Swelling Behavior in Simulated Human Body Fluids and Effect on Macrophages
Source: Polymers (Basel). 2020 Nov 18;12(11):2741. doi: 10.3390/polym12112741 (PMC7699170; doi:10.3390/polym12112741)
Supplement: Supplementary file 1 [file polymers-12-02741-s001.pdf]

polymers

MDPI

Article

Ca-Zn-Ag Alginate Aerogels for Wound Healing Applications: Swelling Behavior in Simulated Human Body Fluids and Effect on Macrophages

Supplementary Material

Claudia Keil [1,*], Christopher Hübner [1], Constanze Richter [1], Sandy Lier [1], Lars Barthel [2], Vera Meyer [2], Raman Subrahmanyam [3], Pavel Gurikov [4], Irina Smirnova [3] and Hajo Haase [1,*]

1 Department Food Chemistry and Toxicology, Institute of Food Technology and Food Chemistry, TU Berlin, Straße des 17. Juni 135, 10623 Berlin, Germany; c.huebner@tu-berlin.de (C.H.); constanze.richter@tu-berlin.de (C.R.); lier@campus.tu-berlin.de (S.L.)

2 Applied and Molecular Microbiology, Institute of Biotechnology, TU Berlin, Straße des 17. Juni 135, 10623 Berlin, Germany; lars.barthel@tu-berlin.de (L.B.); vera.meyer@tu-berlin.de (V.M.)

3 Institute of Thermal Separation Processes, Hamburg University of Technology, Eißendorfer Straße 38, 21073 Hamburg, Germany; raman.subrahmanyam@tuhh.de (R.S.); irina.smirnova@tuhh.de (I.S.)

4 Laboratory for Development and Modelling of Novel Nanoporous Materials, Hamburg University of Technology, Eißendorfer Straße 38, 21073 Hamburg, Germany; pavel.gurikov@tuhh.de

* Correspondence: haase@tu-berlin.de (H.H.); c.keil@tu-berlin.de (C.K.); Tel.: +49 (0) 30 31472701 (H.H.); +49 (0) 30 31472816 (C.K.); Fax: +49 (0) 30 31472823 (C.K.; H.H.)

Received: 23 October 2020; Accepted: 14 November 2020; Published: date

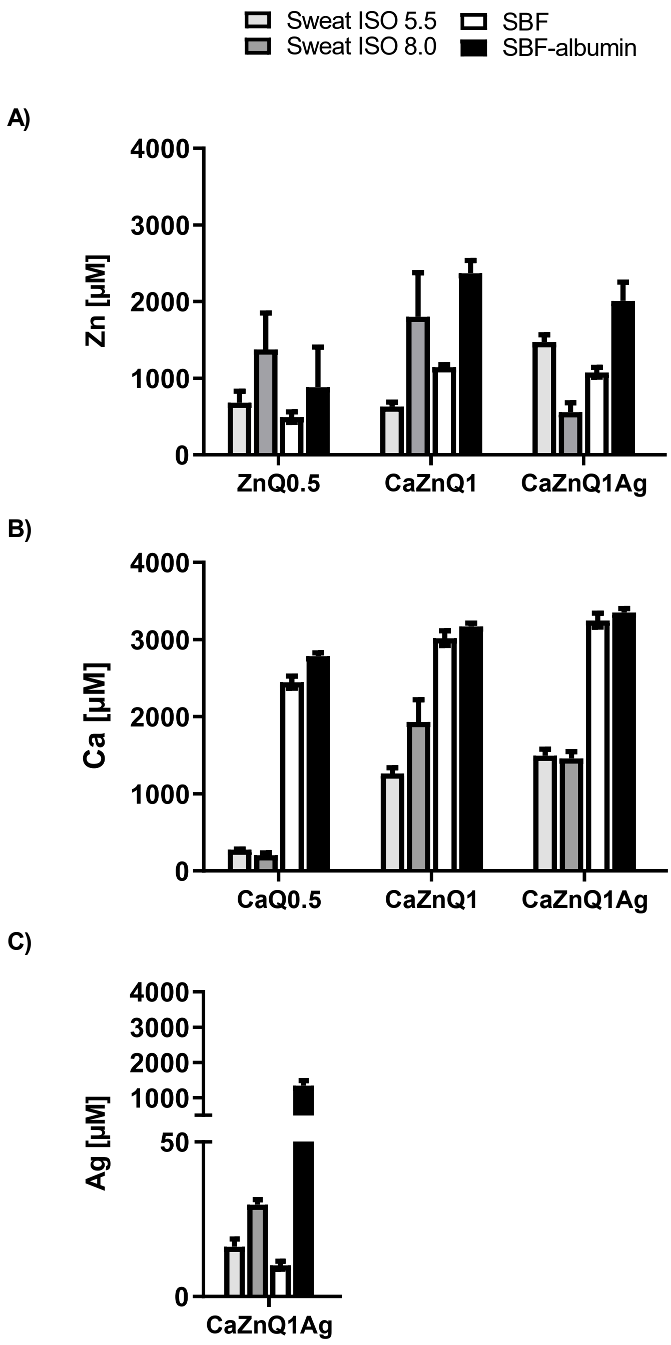

Figure S1. Quantification of total metal concentrations in aerogel swelling supernatants**.** Aerogels were incubated for 5 min in the body fluid substitutes before quantification of the total metal concentrations in the swelling supernatants by flame atomic absorption spectrometry. Data are presented as means ± SEM of three independent experiments.

© 2020 by the authors. Licensee MDPI, Basel, Switzerland. This article is an open access article distributed under the terms and conditions of the Creative Commons Attribution (CC BY) license (http://creativecommons.org/licenses/by/4.0/).
